# Habitat Suitability Assessment for Two Burrowing Rodents on the Island of Lesvos: A Niche-Based Approach

**DOI:** 10.3390/life14101231

**Published:** 2024-09-26

**Authors:** Stylianos P. Zannetos, Konstantinos Theodorou, Yiannis G. Zevgolis, Eleni Galinou, Triantaphyllos Akriotis

**Affiliations:** Biodiversity Conservation Laboratory, Department of Environment, University of the Aegean, University Hill, Mytilene, 81100 Lesvos, Greece; ktheo@aegean.gr (K.T.); zevgolis@env.aegean.gr (Y.G.Z.); elenigalinou@gmail.com (E.G.); takr@aegean.gr (T.A.)

**Keywords:** *Microtus*, *Nannospalax*, Maxent, habitat suitability, fossorial

## Abstract

We conducted a habitat suitability assessment for two burrowing rodents, Anatolian or Nehring’s blind mole rat (*Nannospalax xanthodon*) and Harting’s vole (*Microtus hartingi*), on the island of Lesvos using a niche-based approach. We collected data on the presence of the two species across the island and selected several environmental variables, including land cover, geology, and habitat topography, to assess their influence on habitat suitability. We used the Maxent species distribution modelling algorithm to predict suitable habitats. The results showed that both species preferred habitats with low slopes and specific geological substrates, i.e., alluvial deposits and volcanic rocks. *M. hartingi* showed a preference for open habitats such as saltmarshes and non-irrigated arable land, while *N. xanthodon* preferred non-irrigated arable land, pastures, and discontinuous urban fabric. The model predicted a wider area of suitable habitats for *Microtus hartingi* compared to *N. xanthodon*. Interestingly, the two species are absent from the southeastern part of the island despite our model predicting high suitability; this indicates that a natural barrier of hilly terrain, extensive pine forests, and limestone rock formations may exist that impedes dispersal. Our study provides valuable insights into the habitat preferences of these two burrowing rodents on the island of Lesvos, which can inform biodiversity conservation and ecosystem management strategies.

## 1. Introduction

Anatolian or Nehring’s blind mole rat and Harting’s vole—*Nannospalax xanthodon* (Nordman, 1840) and *Microtus hartingi* (Barrett-Hamilton, 1903), respectively—inhabit parts of the Mediterranean basin. Even though they are both burrowing rodents, there are some important biological differences between them, influencing their own ecological niches. *N. xanthodon* is a strictly subterranean and solitary rodent of the family Spalacidae, one of the three species of the genus *Nannospalax* found in the Mediterranean region [1]. The global distribution of *N. xanthodon* covers mainly the Asiatic part of Turkey, as well as a small area in southwestern Georgia [2], western Armenia [3], and northern Iran [2]. Populations of the species also exist on some northeastern Aegean Islands such as the Greek islands of Lesvos and Limnos [4] and the Turkish islands Gökçeada and Bozcaada [5]. These populations are very important as they represent the westernmost edge of the *N. xanthodon* global range and the only European populations of this species. *N. xanthodon* has a grey pelage and a long cylindrical body of medium–large size. It has weak limbs, lacks a visible neck, and has no external tail [5]. As a solitary species, each individual constructs its own burrow system, which consists of a main tunnel with several secondary pathways branching off [6]. During the digging process, the mole excavates the soil, creating characteristic mounds spaced approximately 25–30 m apart [5]. *N. xanthodon* selects habitats very similar to its congeneric species found in continental Greece, the lesser mole rat (*Nannospalax leucodon* [7]), although it tends to inhabit areas with more xeric conditions [5]. Throughout the Aegean region, optimal habitats for *N. xanthodon* include pastures, orchards, traditional olive groves, and a mosaic of small-scale cultivations [5]. There have been a few reports of *N. xanthodon* damage to agricultural products, particularly to root crops like onion and sugar beet [6], but the species is not categorized as a pest [5]. According to IUCN [7], *N. xanthodon* populations face risks in areas with intensive agriculture and urbanization. Even though the species population is generally stable in suitable habitats, fragmentation, and habitat loss due to human activities pose significant threats to its viability. There is currently limited knowledge regarding the population trends of *N. xanthodon* [7].

On the other hand, *M. hartingi* lives in communal burrow systems and, unlike *N. xanthodon,* it does not spend all of its time in underground burrows. *M. hartingi* is a medium-sized vole with a short tail, typically measuring less than one-third the length of its head and body combined. This species is distributed across the Balkan Peninsula, including Northern Macedonia, the southernmost regions of Serbia, Greece, Bulgaria, and significant parts of Turkey [8]. Even though the species occurs in structurally simple habitats, the species’ distribution across Europe is remarkably fragmented [9]. Lesvos is the only mediterranean island hosting a population of a social vole [10,11] and one of only three islands inhabited by Microtus species in the entire Mediterranean basin. Harting’s vole is commonly found in well-drained meadows, on barren hill slopes with scattered trees and occasionally in agricultural fields like alfalfa (S.P. Zannetos, personal observation). Nevertheless, its presence in agricultural fields is temporary, as it shallow nests and tunnel systems are easily destroyed by ploughing. Moreover, *M. hartingi* burrow systems are commonly found along riverbanks and marshlands (personal observation) [8]. Along the Aegean coast and on Lesvos, burrow systems can also be found in traditional olive groves (S.P. Zannetos, personal observation). *M. hartingi* population densities can increase dramatically in some cases, leading to significant losses for farmers [12].

For our goal, species distribution modelling (SDM) is an appropriate and widely used approach for simulating a species’ ecological niche. It also provides valuable ecological insights into the environmental factors influencing species distribution and assists in pinpointing potential habitats for species occurrence. Over recent years, several effective SDM techniques have emerged, such as generalized linear and additive models, Ecological Niche Factor Analysis, and random forest [13]. These models have proven their reliability across a broad range of applications globally, effectively predicting habitat suitability for numerous species [14,15,16,17]. In this study, we employed the maximum-entropy (Maxent) species distribution modelling algorithm, noted for its strong predictive capabilities, as demonstrated by its application in over 1000 ecological studies [16].

## 2. Materials and Methods

### 2.1. Study Area

The island of Lesvos is in the northeastern Aegean Sea, very close to the coastline of western Turkey, and covers an area of 1633 km^2^, being the third largest island in the Aegean Sea and the eighth largest in the Mediterranean. Lesvos has a rough, mountainous terrain, with its highest peak (Mount Lepetymnos) rising to an elevation of 968 m. The eastern and southern parts of the island have a heterogeneous landscape, including traditional olive groves [18], pastures, and a variety of non-intensive agricultural crops on flat land. The central part of the island is characterized by an extensive continuous pine (*Pinus brutia*) forest [19], while the western and northern parts have a markedly different landscape, with low shrubby vegetation, known as ‘phrygana’, along with patches of oak woodland and small patched of non-intensive agriculture. The island’s climate is Mediterranean with hot, dry summers, and cool, wet winters. The mean monthly temperature is 9.6 °C in January, the coolest month, and 27.0 °C in July, the hottest month. Total annual precipitation is 645 mm at Mytilini Airport (1955–2010) [20], with a total of only 23.5 mm during the driest months (June to September).

### 2.2. Field Collection Data

**Figure 1:**
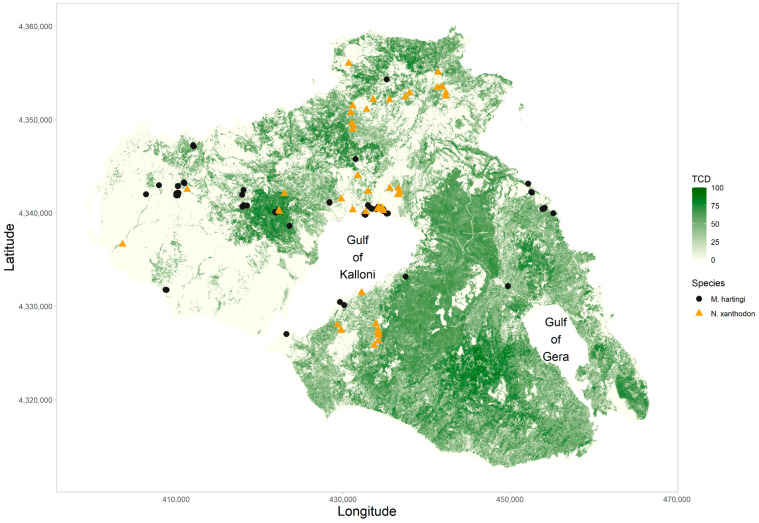
Field observations of *M. hartingi* (circles) and *N. xanthodon* (triangles) on the island of Lesvos. The background shading depicts Tree Cover Density (TCD) as a continuous gradient between 0 and 1.

During the years 2023 and 2024, we conducted a survey of *M. hartingi* and *N. xanthodon* across Lesvos. We collected data at a total of 112 presence locations (Figure 1); of these, 65 were occupied by *M. hartingi* and 47 were occupied by *N. xanthodon*. Presence was defined as either observation of live animals or detection of either burrows in the case of *M. hartingi* or of the distinctive soil mounds in the case of *N. xanthodon*.

**Figure 2:**
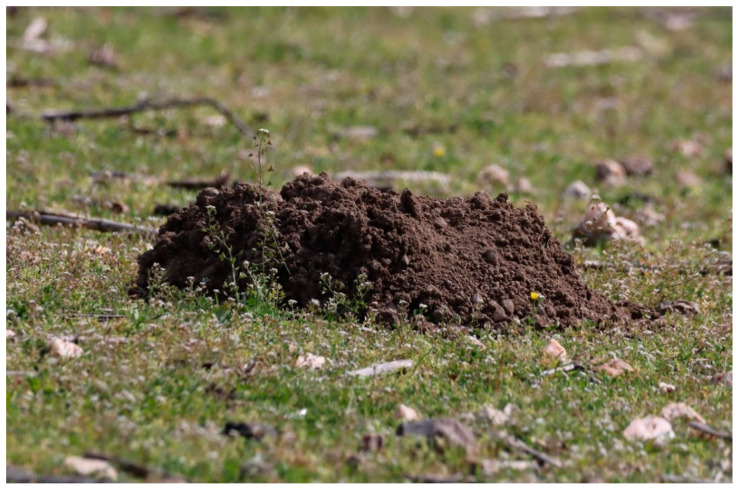
Characteristic soil mound of *N. xanthodon* in Lesvos, Greece.

**Figure 3:**
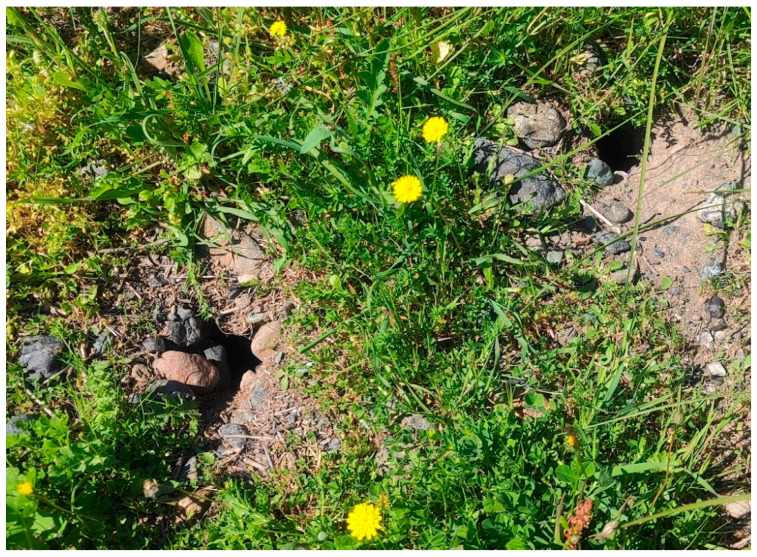
*M. hartingi* burrow entrances in Kalloni salt pans (March 2024).

Our efforts were concentrated between January and the end of May for *N. xanthodon*, matching the breeding period of this species, during which animal activity increases (S.P. Zannetos, personal observation). The excavated soil mounds (see Figure 2) of *N. xanthodon* could easily be identified even from a distance. To avoid redundancy in our data, we adhered to a consistent minimum distance of 100 m between the soil mounds attributed to each individual *N. xanthodon*. We focused on *M. hartingi* from May to November, when burrow holes are more visible due to the vegetation gradually drying up and becoming shorter and less dense. *M. hartingi* burrows were identified by their numerous uncovered entrance holes (see Figure 3), having an average diameter of 4 to 7 cm (S.P. Zannetos, unpublished data).

This characteristic, combined with the fact that *M. hartingi* is the only rodent with communal burrow systems on Lesvos, as well as the audible squeaking sounds emitted by the voles from their burrows, aided in their identification.

### 2.3. Environmental Predictors

To assess the habitat suitability of the two study species, several environmental variables were chosen, based on their possible importance in influencing the habitat selection of *M. hartingi* and *N. xanthodon*. The prediction variables used are the following:

**Land Cover variables**: We used the categorisation made by the CORINE Land Cover (CLC) inventory [21] and the Tree Cover Density (TCD) subset of the COPERNICUS high resolution layer [22], representing the percentage of tree cover derived from the CORINE inventory. CLC category ‘natural grasslands’ in Lesvos actually corresponds to areas covered by low xerophytic shrubby vegetation. This type of vegetation is called ‘phrygana’ and we use this term instead because ‘grasslands’ is misleading.

**Figure 4:**
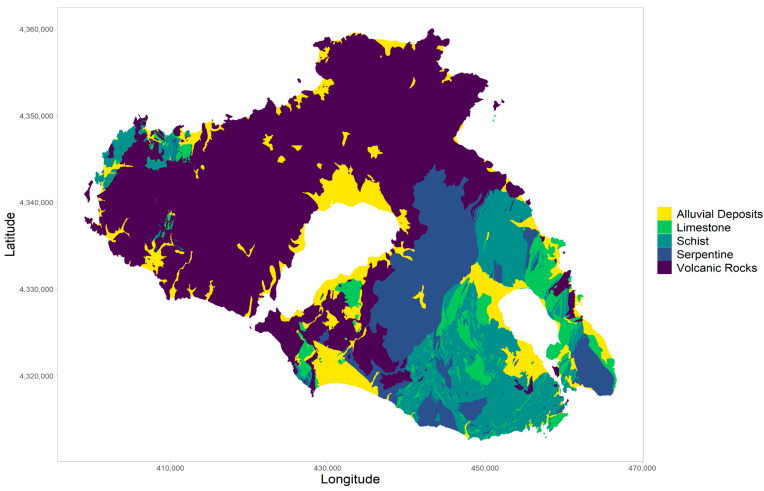
Simplified geological map of Lesvos. Source: [23]. We grouped the diverse range of geological formations into five categories: (1) limestone, (2) schist, (3) alluvial deposits, (4) serpentine, and (5) volcanic rocks.

**Geology**: We used a simplified classification of the island’s geological structure, derived from the geological map shown in Figure 4 [23]. The classification groups the diverse range of geological formations into five categories: (1) limestone, (2) schist, (3) alluvial deposits, (4) serpentine, and (5) volcanic rocks. This geological classification was used as a proxy for soil properties.

**Habitat topography variables**: To represent the habitat topography we included the following variables: (a) slope, (b) elevation, and (c) aspect. Aspect values were simplified to the north–south component, ranging from 0° (due north) to 180° (due south).

All predictor variables were cropped to the same extent on the map and resampled to a resolution of 10 m × 10 m. To address collinearity issues, we performed a variance inflation factor (VIF) analysis on the predictor variables [24]. All variables were retained based on a correlation threshold of r = 0.8.

### 2.4. Species Distribution Modelling (SDM) and Validation

Species distribution modelling was performed in R (version 4.3.3) with the SDMtune package, which implements the Maxent algorithm. Maxent is a powerful machine learning algorithm that aims to estimate the most uniform distribution (maximum entropy) across the study area, while ensuring that the expected value of each environmental variable under this estimated distribution matches its empirical average. This approach often outperforms other presence-only modelling techniques [25]. We generated 500 random background points, which is about ten times the number of observations of each species. Subsequently, we used the thin() function from the spThin package to thin both presence and background points, avoiding spatial biases [26]. This process excluded points closer than 100 m from each other, resulting in 64 presence points for M. hartingi, 47 points for N. xanthodon, and approximately 400 absence points. The datasets were split into two parts, with 80% of the points used to train the model and 20% of the points to evaluate it.

While training and evaluation data ideally come from independent datasets [27], ours originates from a single dataset. To address potential spatial autocorrelation, we applied spatial blocking using the R package blockCV [28]. The function cv_spatial_autocor() within this package determined optimal block sizes of 3900 m and 887 m for M. hartingi and N. xanthodon, respectively. The higher block size for Microtus hartingi could indicate higher home range and movement patterns, spatial autocorrelation in environmental preferences or higher population density and clustering.

Thus, we built random square spatial blocks of the corresponding sizes and split the data into five spatially separated folds. By filtering the data and implementing spatial blocking, we aimed to address model overfitting, which could reduce the predictive power of our results [29].

We used the final model for the following purposes: (i) to predict potentially suitable habitats for the two species; (ii) to assess the relative importance of environmental variables in determining habitat suitability using their permutation importance and response curves; (iii) to evaluate model performance using the AUC criterion (area under the receiver operating characteristic (ROC) curve) and the True Skill Statistic (TSS). AUC quantifies the probability that the model correctly ranks a random presence locality higher than a random background pixel. Its advantages include being independent of prevalence and providing a single measure of model performance, eliminating the need to choose a threshold [26]. AUC ranges from 0 to 1, with higher values indicating better model performance, while values <0.5 show that the model is no better than random.

TSS, on the other hand, is a threshold-dependent metric that measures the ability of a model to correctly predict presence and absence. It is calculated as TSS = sensitivity + specificity − 1, where sensitivity (also known as the true positive rate) measures the proportion of actual positives correctly identified, and specificity (also known as the true negative rate) measures the proportion of actual negatives correctly identified. TSS ranges from −1 to 1, where a score of 1 indicates perfect agreement between observed and predicted classifications, 0 indicates a performance no better than random, and negative values indicate performance worse than random. This makes TSS a valuable tool for evaluating the predictive accuracy of models, as it considers both the [30] presence and absence predictions, providing a single measure that balances the two. As a general guideline, TSS values above 0.5 are commonly considered to indicate good model performance [31]. It has been shown that the minimisation of the difference between sensitivity and specificity is one of the most reliable methods in order to produce a presence–absence map [15]. We, thus, estimated binary habitat suitability using a cut-off threshold based on equal sensitivity and specificity.

## 3. Results

### 3.1. Significant Explanatory Variables and Model Performance

**Figure 5:**
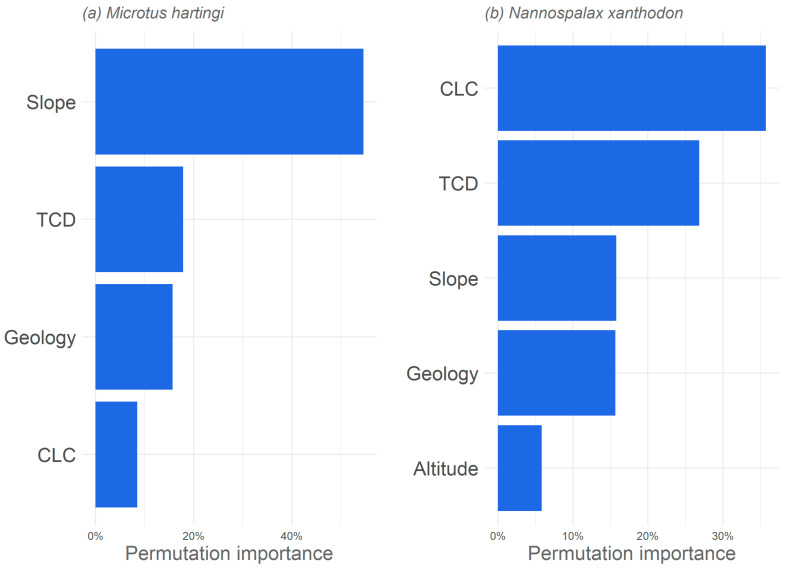
The relative importance of environmental variables in determining habitat suitability using their permutation importance for (**a**) *M. hartingi* and (**b**) *N. xanthodon*. Only variables with a contribution higher than 5% are presented.

The models retained seem to adequately simulate the distribution of species under study, with an AUC (TSS) ranging from 0.86 (0.62) for the case of *M. hartingi* to 0.91 (0.69) for *N. xanthodon*. Figure 5 summarises the relative importance of each environmental predictor in influencing the distribution of the two species. Permutation importance revealed CLC as the most critical factor for the model of *N. xanthodon*, exceeding a 35% contribution. TCD also played a significant role for this species with more than 25% contribution. In contrast, slope was the most influential variable (35%) for *Microtus hartingi*, while CLC had the least influence with 5% contribution. Interestingly, geology consistently influenced both species (18% for *M. hartingi* and 16% for *N. xanthodon*). Additionally, *N. xanthodon* distribution was slightly affected by altitude, with a contribution of less than 10%.

### 3.2. Response of the Study Species to Environmental Variables

**Figure 6:**
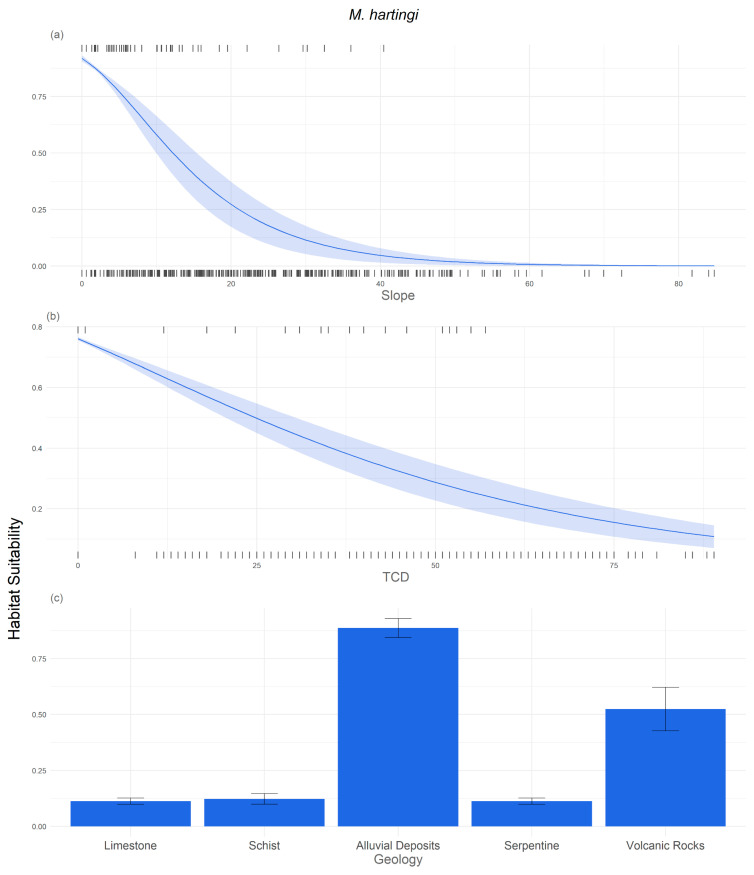

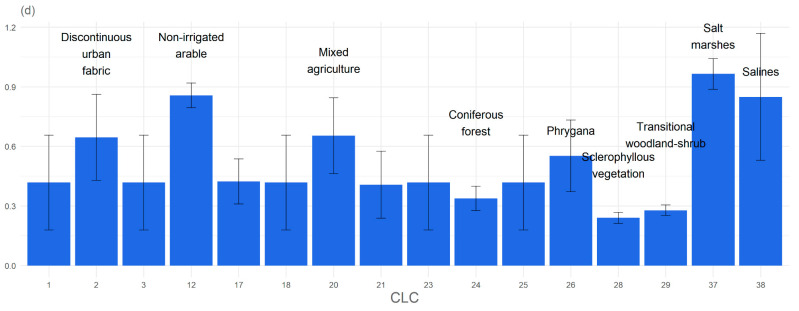
Response curves of habitat suitability to environmental variables: (**a**) slope, (**b**) Tree Cover Density (TCD), (**c**) geology, and (**d**) CORINE Land Cover (CLC). These were the variables with a permutation importance higher than 5% according to Maxent model for *M. hartingi*. We annotated the most and least favourable CLC categories for better understanding.

**Figure 7:**
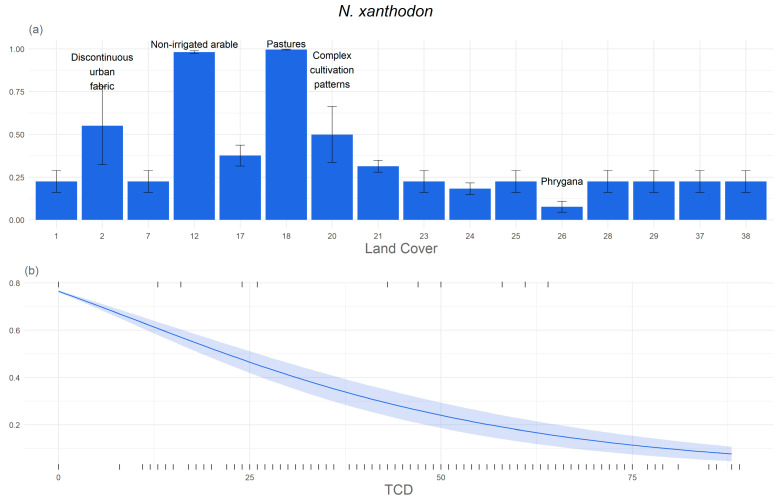

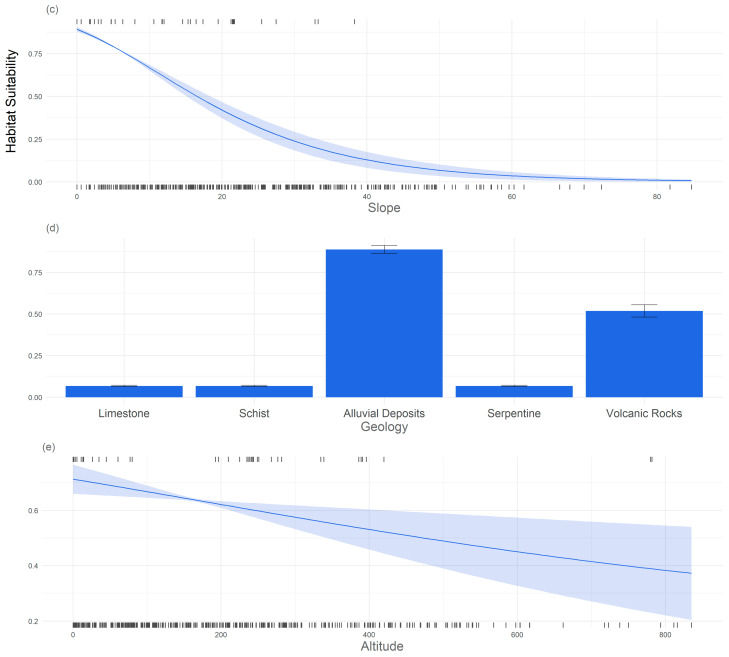
Response curves of habitat suitability to environmental variables: (**a**) CORINE Land Cover (CLC), (**b**) Tree Cover Density (TCD), (**c**) slope, (**d**) geology, and (**e**) altitude. These were the variables with a permutation importance higher than 5% according to Maxent model for *N.xanthodon*. We annotated the most and least favourable CLC categories for better understanding.

The most important response curves of the two species to environmental variables are presented in Figure 6 and Figure 7. These response curves show how each environmental variable affects the model predictions. Analysis of the response curve in Figure 6 shows that steeper slopes have a negative impact on habitat suitability for *M. hartingi*, suggesting that flat areas are more favourable for burrow excavation in this species. Additionally, TCD adversely affects habitat suitability, indicating that habitats with less dense tree cover are preferable for the voles. It is noteworthy that areas where habitat suitability exceeds 60% (Figure 6) tend to have sparse tree cover or no trees, further emphasizing the preference of *M. hartingi* for open habitats such as saltmarshes, phrygana, and non-irrigated arable land. The least preferred land cover categories for *M. hartingi* include coniferous forests, sclerophyllous vegetation, and transitional woodland–shrub areas, all with a habitat suitability of less than 30%.

For *N. xanthodon*, the most suitable land cover categories, with habitat suitability exceeding 50%, include non-irrigated arable land, pastures, discontinuous urban fabric, and areas with complex cultivation patterns. Conversely, phrygana exhibit the lowest habitat suitability, with less than 15%. Similarly to *M. hartingi*, both TCD and slope demonstrate a negative relationship with habitat suitability for *N. xanthodon*. This is further supported by the result for altitude, with suitability decreasing towards higher altitude. In terms of geology, alluvia and volcanic rocks exhibit the highest habitat suitability, with 75% and 50%, respectively.

## 4. Discussion

Both species prefer habitats which are characterized by low slopes and specific geological substrate. *M. hartingi* shows a clear preference for open habitats, which in the case of Lesvos include saltmarshes, the area around the two human-made salines (Kalloni and Polichnitos saltpans) and non-irrigated arable land, avoiding taller and denser woody vegetation such as sclerophyllous vegetation, transitional woodland, and mature coniferous forests. In general terms, our results agree with the existing literature [8,32]. In a few cases we found *M. hartingi* in unusual situations, such as in clearings within extensive coniferous forest (*Pinus brutia* and *Pinus nigra*) in western Lesvos above the village of Parakoila (C. Sazeidis, personal observation), as well as in a seasonally flooded agricultural area, known as Megali Limni, in the central continuous coniferous forest of the island. This indicates a significant degree of adaptability to anthropogenically altered or naturally patchy landscapes. On the other hand, *N. xanthodon* is preferentially associated with non-irrigated arable land, pastures, and discontinuous urban fabric, which, in the case of Lesvos, translates to scattered holiday homes or rural dwellings surrounded by small-scale cultivation. In contrast to *M. hartingi*, our model indicates that phrygana would be unsuitable for *N. xanthodon*. Although phrygana would be expected to be rather similar to pastures, a highly preferred land cover type, it is likely that the soil is too shallow and stony, as well as occurring in small patches [33], making phrygana unsuitable for the larger *N. xanthodon* as compared with the smaller vole. Further, Lesvos phrygana are invariably dominated by *Sarcopoterium spinosum* (S.P. Zannetos, personal observation) and avoidance of *S. spinosum* cover has also been reported for the blind mole rat *N. galili* in Israel [34]. Furthermore, our model suggests that both species prefer alluvial soils and volcanic rock substrate, avoiding limestone, schist, and serpentine substrates. *M. hartingi* is known to avoid shallow skeletal soils [35] and to prefer mollisols and entisols [32], such as those over limestone and serpentine substrates on Lesvos, while *N. xanthodon*, as well as *N. leucodon* and *N. ehrenbergi*, are all reported to avoid shallow soils over rocky substrate and to prefer sands, loess, loam and rendzina [1]. This could be due to several factors, including food availability and suitability for burrowing. Alluvial and volcanic rock areas offer more abundant or accessible food resources as they can support a wide variety of plant life, which could provide different food sources all year around, with both species feeding on vegetative plant parts [36,37]. In contrast, soils over serpentine are well known for their high levels of toxic metals like nickel and chromium and low levels of nutrients, with lower vegetation productivity, lower plant diversity, and possible toxicity to animals as well as plants [38]. Alkaline soils over limestone might also limit the availability of certain nutrients and, further, although they facilitate high plant diversity, they have a shorter growth period due to drier soil conditions. The physical properties of soils in alluvial and volcanic areas appear to be more suitable for burrowing than the rocky and often shallow soils over limestone and schist. Volcanic substrates, although variable, are often friable, producing deep soils suitable for fossorial rodents.

**Figure 8:**
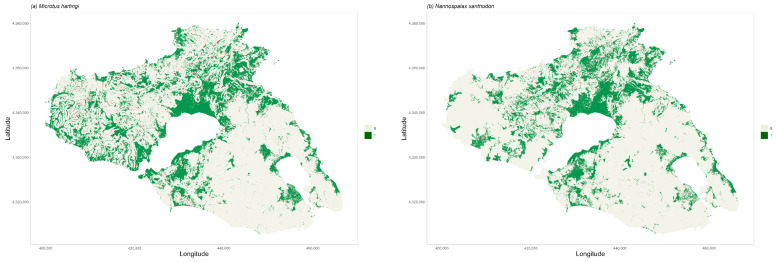
Binary habitat suitability by applying a cut-off threshold based on equal sensitivity and specificity for (**a**) *M. hartingi* and (**b**) *N. xanthodont*, predicted by the Maxent model.

Comparing habitat suitability of the two species predicted by the Maxent model, *M. hartingi* appears to have a wider area of suitable habitats across the island. The binary model indicates that approximately 34% of the island’s land area is potentially suitable for *M. hartingi*, compared to 22% for *N. xanthodont*, as can be seen in Figure 8. While such results should be approached with caution and may not fully represent the actual distribution of the species or actual habitat suitability, they do suggest niche breadth differences between the two species. This is brought about by the dependence of *N. xanthodon* on a wider array of environmental variables, meaning that a more specific combination of conditions must be satisfied for an area to be suitable for the species, compared with *M. hartingi*. Moreover, the model predicts a more limited selection of favourable land cover types for *N. xanthodon*, further underscoring its more specialised habitat requirements.

Interestingly, although our model predicts habitats with high suitability in the southeastern part of the island, particularly around the Gulf of Gera, both species appear to be completely absent from this area. There are no records of the species’ historical distribution on the island. Therefore, we cannot know whether they were once present in the southeastern part of the island and later disappeared due to human influence. Such an interpretation seems unlikely as human activities such as agriculture are not intensive in this part of the island, and there is no indication that such activities have ever been more intesnsive in the area compared with the rest of the island, where the two species.An alternative explanation for the absence of both species from the southeastern part of the island could stem from the natural barrier of the intervening hilly massif which combines steep slopes, extensive pine forest, and limestone rock. Our model shows that these characteristics constitute an unfavourable habitat and are likely to impede dispersal for both species. Thus, these areas of suitable habitat in the southeastern part of the island appear to be isolated fragments where the species cannot survive in the long term.

The findings of this study will inform biodiversity conservation and ecosystem management strategies on the island of Lesvos, contributing to evidence-based conservation efforts aimed at preserving the habitats of these fossorial rodents and promoting their sustainable coexistence with human activities on the island.

## 5. Conclusions

Land cover, Tree Cover Density, slope, and geological substrate are important environmental variables in determining habitat suitability for both *M. hartingi* and *N. xanthodon* on the island of Lesvos. Both slope and Tree Cover Density are negatively connected to habitat suitability, suggesting a preference for open flat or gently sloping land. Non-irrigated arable land and pastures are the most favourable CLC categories for *N. xanthodon*, in which CLC is the most important environmental variable, with TCD also making a high contribution, being second in importance in the model obtained for this species. Habitat suitability for *M. hartingi* is more influenced, negatively, by slope and much less by other variables, with little differentiation between CLC categories. Both species are absent from southeastern Lesvos, where suitable habitat is patchy and isolated and where neither species can maintain populations in the long term. Our study is the first to address habitat suitability for the island populations of these two species. Further work could clarify the effect of geological substrate and land cover with respect to food availability and soil properties affecting burrow excavation.

## Data Availability

The datasets generated during and/or analysed during the current study are available from the corresponding author on reasonable request.
